# Relationships among Postprandial Plasma Active GLP-1 and GIP Excursions, Skeletal Muscle Mass, and Body Fat Mass in Patients with Type 2 Diabetes Treated with Either Miglitol, Sitagliptin, or Their Combination: A Secondary Analysis of the MASTER Study

**DOI:** 10.3390/jcm12093104

**Published:** 2023-04-24

**Authors:** Masahiro Sato, Hiroki Fujita, Hiroki Yokoyama, Atsushi Mikada, Yohei Horikawa, Yuya Takahashi, Yuichiro Yamada, Hironori Waki, Takuma Narita

**Affiliations:** 1 Department of Metabolism and Endocrinology, Akita University Graduate School of Medicine, Akita 010-8543, Japan; 2 Jiyugaoka Medical Clinic, Obihiro 080-0016, Japan; 3 Gastroenterology and Diabetes Unit, Hiraka General Hospital, Yokote 013-8610, Japan; 4 Center for Diabetes, Endocrinology and Metabolism, Kansai Electric Power Hospital, Osaka 553-0003, Japan; 5 Akita Higashi Medical Clinic, Akita 010-0041, Japan

**Keywords:** active GLP-1, active GIP, skeletal muscle mass, body fat mass, miglitol, sitagliptin, type 2 diabetes

## Abstract

Background: We previously conducted a pilot randomized controlled trial “the MASTER study” and demonstrated that alpha-glucosidase inhibitor miglitol and a dipeptidyl peptidase-4 inhibitor sitagliptin modified postprandial plasma excursions of active glucagon-like peptide-1 (aGLP-1) and active gastric inhibitory polypeptide (aGIP), and miglitol treatment decreased body fat mass in patients with type 2 diabetes (T2D). However, the details regarding the relationships among postprandial plasma aGLP-1 and aGIP excursions, skeletal muscle mass, and body fat mass are unclear. Methods: We conducted a secondary analysis of the relationships among skeletal muscle mass index (SMI), total body fat mass index (TBFMI), and the incremental area under the curves (iAUC) of plasma aGLP-1 and aGIP excursions following mixed meal ingestion at baseline and after 24-week add-on treatment with either miglitol alone, sitagliptin alone, or their combination in T2D patients. Results: SMI was not changed after the 24-week treatment with miglitol and/or sitagliptin. TBFMI was reduced and the rates of aGIP-iAUC change were lowered in the two groups treated with miglitol, although their correlations did not reach statistical significance. We observed a positive correlation between the rates of aGIP-iAUC and TBFMI changes and a negative correlation between the rates of TBFMI and SMI changes in T2D patients treated with sitagliptin alone whose rates of aGIP-iAUC change were elevated. Conclusions: Collectively, although T2D patients treated with miglitol and/or sitagliptin did not show altered SMI after 24-week treatment, the current study suggests that there are possible interrelationships among postprandial plasma aGIP excursion modified by sitagliptin, skeletal muscle mass, and body fat mass.

## 1. Introduction

The primary physiologic role of the two main incretin hormones glucagon-like peptide-1 (GLP-1) and gastric inhibitory polypeptide (GIP) is a potentiation of glucose-dependent insulin secretion from pancreatic β-cells [1,2]. GLP-1 is released from L-cells located broadly from the upper intestine to the colon [3], whereas GIP is secreted from K-cells that are located in the proximal part of the small intestine, duodenum, and jejunum [1]. These incretin hormones are released in response to the ingestion of nutrients including glucose, fatty acids, and essential amino acids [2,4,5], and rapidly degraded and inactivated by dipeptidyl peptidase-4 (DPP-4) [1,6,7]. Therefore, DPP-4 inhibitors exert a glucose-lowering effect by enhancing active incretin hormones in circulating plasma. Indeed, DPP-4 inhibitors such as sitagliptin have been demonstrated to markedly reduce DPP-4 activity in mouse plasma [8]. On the other hand, alpha-glucosidase inhibitors (αGIs) delay complex carbohydrate digestion and intestinal glucose absorption by competitive inhibition of the enzyme α-glucosidase at the brush border of the small intestine, and thereby reduce postprandial plasma glucose and insulin levels [9]. Our clinical studies and others have previously reported that an αGI miglitol suppresses GIP release and increases GLP-1 secretion after meal ingestion [10,11,12,13]. Since miglitol is almost completely absorbed in the upper part of the small intestine [14], it would suppress glucose absorption in the area where GIP-secreting K-cells are located, thereby reducing GIP secretion after meal ingestion. In addition, through the above-mentioned mechanism, intestinal glucose absorption would be shifted to the lower part of the small intestine, and thereby glucose-stimulated GLP-1 secretion from L-cells may be enhanced. Thus, postprandial plasma GLP-1 and GIP excursions are highly affected by DPP-4 inhibitors and αGIs.

Recently, we have demonstrated that GLP-1 and GIP receptors are expressed in multiple organs through mRNA expression analysis in mice [15], although the expression of their receptors has not been fully investigated in humans. According to the experimental data, GIP receptors are moderately expressed in muscle tissues, whereas GLP-1 receptors are expressed, to a lesser extent, in muscle tissues [15]. Indeed, GLP-1 and GIP receptor signaling has been reported to exert various extra-pancreatic actions [1,16]. For example, in our experimental studies, GLP-1 receptor signaling has been demonstrated to provide a protective effect against oxidative stress in diabetic mouse kidneys [17] and to reduce the pathogen load in mice with experimental influenza virus infection via increased expression of intracellular interferon-inducible GTPases [18], as extra-pancreatic actions.

Although the roles of incretin hormones on muscle regeneration, fat accumulation, or their metabolism have been explored in clinical and experimental studies, it is unclear whether postprandial plasma active GLP-1 (aGLP-1) and active GIP (aGIP) excursions modified by treatment with DPP-4 inhibitors and/or αGIs affect the regulation of skeletal muscle mass and body fat mass. Therefore, we conducted a secondary analysis of data on skeletal muscle mass index (SMI), total body fat mass index (TBFMI), and the incremental area under the curves (iAUC) of plasma aGLP-1 and aGIP excursions, following mixed meal ingestion at baseline and after 24-week add-on treatment, with an αGI miglitol and/or a DPP-4 inhibitor sitagliptin in patients with type 2 diabetes (T2D) from the “Miglitol and Sitagliptin on gastric inhibitory polypeptide secretory responses in T2D patients with obesity” study (MASTER study) [19].

## 2. Materials and Methods

### 2.1. Study Design

The MASTER study [19] was a multicenter, randomized, parallel, open, three-armed, 24-week pilot study to assess the changes in plasma active and total levels of GLP-1 and GIP following mixed meal (460 kcal containing 56.5 g carbohydrates) ingestion at baseline and after the add-on treatment with either miglitol, sitagliptin, or a combination with miglitol plus sitagliptin for 24 weeks in Japanese T2D patients receiving diet therapy alone or taking metformin and/or sulfonylurea. Of the 49 T2D patients enrolled in the study from July 2011 to June 2012, 47 patients were randomly assigned to three treatment groups; miglitol (*n* = 15), sitagliptin (*n* = 16), and a combination with miglitol plus sitagliptin (*n* = 16). Finally, a total of 41 patients aged 40–78 years and treated with either miglitol (*n* = 14), sitagliptin (*n* = 14), or a combination with miglitol plus sitagliptin (*n* = 13) completed the study and their data were analyzed. In the present study, we performed a secondary analysis of data regarding SMI, TBFMI, iAUC of plasma aGLP-1 excursion (aGLP-1-iAUC), iAUC of plasma aGIP excursion (aGIP-iAUC), iAUC of plasma glucose excursion (glucose-iAUC), and iAUC of serum immunoreactive insulin (IRI) excursion (IRI-iAUC) following mixed meal ingestion at baseline and after 24-week add-on treatment with miglitol and/or sitagliptin in 35 T2D patients aged 40–78 years, whose SMI values were available in the MASTER study. The final sample consisted of 11 (9 males and 2 females), 13 (10 males and 3 females), and 11 (6 males and 5 females) T2D patients treated with miglitol, sitagliptin, or a combination with miglitol plus sitagliptin for 24 weeks, respectively. This study was approved by the Ethics Committees of Akita University (protocol code 2328, November 2019), and was performed in accordance with the Declaration of Helsinki. The MASTER study was registered in the University Hospital Medical Information Network (UMIN) clinical trials registry system (trial ID 000006098). Written informed consent was obtained from all participants prior to their participation.

### 2.2. Measurements of Body Composition

Appendicular skeletal muscle mass (ASM) and total body fat mass (TBFM) were determined by bioelectrical impedance analysis using X-Scan plus (Jawon Medical, Kungsan, Republic of Korea). SMI (kg/m^2^) and TBFMI (kg/m^2^) were calculated as ASM and TBFM divided by height squared (m^2^), respectively. The rates of SMI, TBFMI, aGLP-1-iAUC, aGIP-iAUC, glucose-iAUC, and IRI-iAUC changes following mixed meal ingestion (%) were calculated as ([the value after 24-week treatment] − [the value at baseline])/(the value at baseline) × 100. We assessed the relationships between the rates of SMI, TBFMI, aGLP-1-iAUC, aGIP-iAUC, and IRI-iAUC changes.

### 2.3. Statistical Analysis

Differences between the values at baseline and after the treatment were analyzed using the Wilcoxon signed rank test. Differences among the three groups were determined by the Kruskal-Wallis test, followed by Dunn’s multiple comparison test and chi-square test. Pearson’s correlation coefficient was calculated to measure the strength and direction of a linear association between the two rates of changes. Simple linear regression was used to model the relationship between the two rates of changes, calculate the coefficient of determination, and construct the best fit line. A value of *p* < 0.05 was considered statistically significant. Statistical analysis was performed using GraphPad Prism 9 software (GraphPad, San Diego, CA, USA).

## 3. Results

### 3.1. Clinical Characteristics and Physiological and Biochemical Parameters

The proportion of males and females, age, body weight, body mass index, systolic and diastolic blood pressure, fasting plasma glucose, fasting serum IRI, HbA1c, LDL-cholesterol, HDL-cholesterol, triglyceride, fasting plasma active GLP-1 and GIP, and the proportion of patients treated with metformin, sulfonylurea, antihypertensive agents, and lipid-lowering agents were not significantly different among the three groups treated with miglitol, sitagliptin, and a combination with miglitol plus sitagliptin at baseline (Table 1). Fasting plasma glucose after 24-week treatment was significantly reduced in the miglitol plus sitagliptin-treated group, and HbA1c after 24-week treatment was significantly lowered in the two groups treated with sitagliptin and a combination with miglitol plus sitagliptin (Table 1). Fasting plasma active GLP-1 and GIP were significantly elevated after 24-week treatment in the two groups treated with sitagliptin and a combination with miglitol plus sitagliptin (Table 1).

### 3.2. Changes in SMI and TBFMI

**Figure 1:**
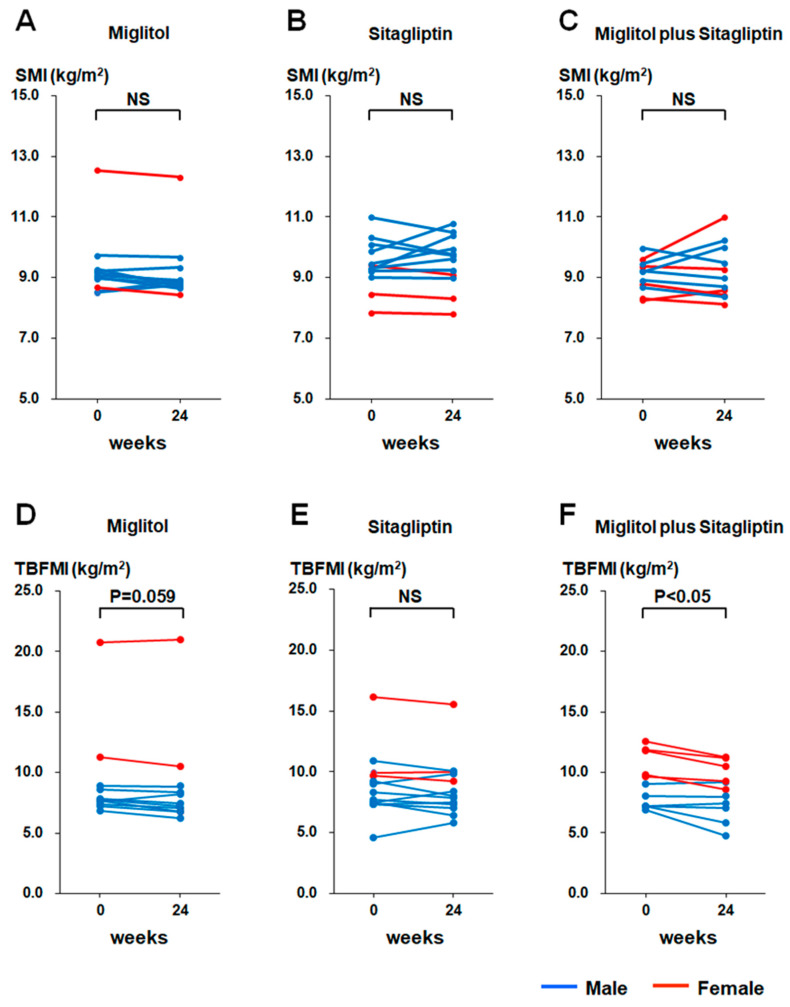
Changes in skeletal muscle mass index (SMI) and total body fat mass index (TBFMI) from baseline (week 0) to the end of 24-week treatment with miglitol, sitagliptin, and a combination with miglitol plus sitagliptin. (**A**,**D**), group treated with miglitol alone; (**B**,**E**), group treated with sitagliptin alone; (**C**,**F**) group treated with a combination of miglitol plus sitagliptin; NS, not significant.

SMI was not significantly different between baseline and the end of 24-week treatment in the three groups treated with miglitol, sitagliptin, and a combination with miglitol plus sitagliptin (Figure 1A–C). Furthermore, a 24-week decrease in SMI was not observed in the three groups (Figure 1A–C). The Asian Working Group for Sarcopenia (AWGS) 2019 consensus specified the cutoffs for height-adjusted muscle mass in bioimpedance for sarcopenia as <7.0 kg/m^2^ in men and <5.7 kg/m^2^ in women, and also defined sarcopenia as age-related loss of muscle mass, plus low muscle strength (handgrip strength <28 kg in men and <18 kg in women), and/or low physical performance (6-m walk < 1.0 m/s, Short Physical Performance Battery score ≤ 9 points, or 5-time chair stand test ≥ 12 s) [20]. According to the diagnostic criteria of sarcopenia, the patients with sarcopenia were not included in this study. TBFMI tended to be reduced at the end of 24-week treatment with miglitol relative to baseline (*p* = 0.059, Figure 1D), and was significantly decreased at the end of 24-week treatment with miglitol plus sitagliptin relative to baseline (*p* < 0.05, Figure 1F), whereas a significant change in TBFMI was not observed after the 24-week treatment with sitagliptin (Figure 1E).

### 3.3. Rates of SMI, TBFMI, aGLP-1-iAUC, aGIP-iAUC, Glucose-iAUC, and IRI-iAUC Changes

**Figure 2:**
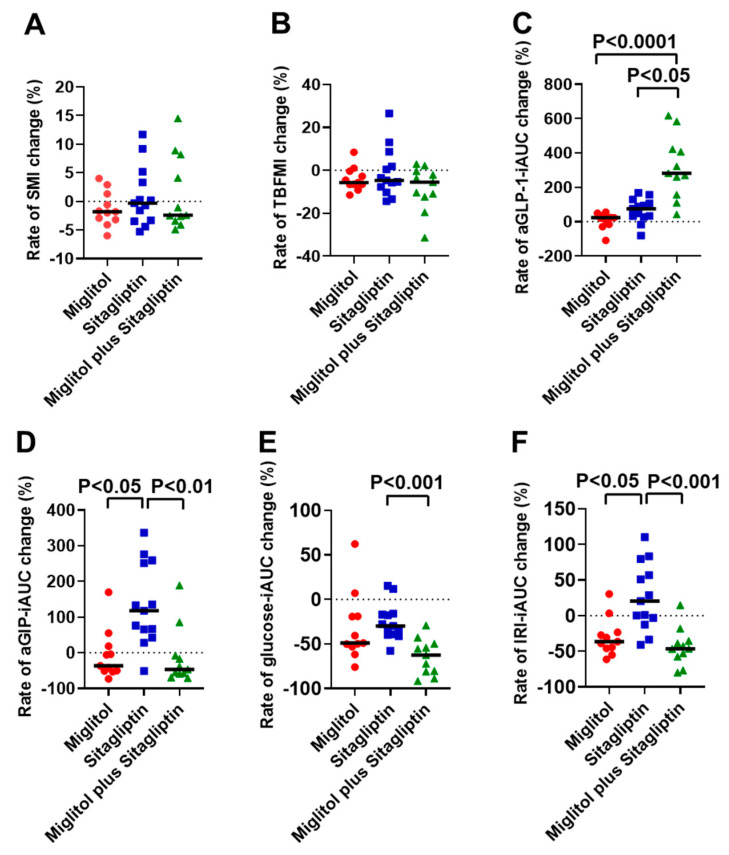
Rates of skeletal muscle mass index (SMI), total body fat mass index (TBFMI), aGLP-1-iAUC, aGIP-iAUC, glucose-iAUC, and IRI-iAUC changes from baseline to the end of 24-week treatment with miglitol, sitagliptin, and a combination with miglitol plus sitagliptin. (**A**) rate of SMI change; (**B**) rate of TBFMI change; (**C**) rate of aGLP-1-iAUC change; (**D**) rate of aGIP-iAUC change; (**E**) rate of glucose-iAUC change; (**F**) rate of IRI-iAUC change. Bars indicate median.

Significant differences in the rates of SMI and TBFMI changes from baseline to the end of 24-week treatment were not observed among the three groups treated with miglitol, sitagliptin, and a combination with miglitol plus sitagliptin (Figure 2A,B). The majority of patients treated with miglitol alone and a combination with miglitol plus sitagliptin exhibited a reduction in TBFMI (Figure 2B). The rate of aGLP-1-iAUC change from baseline to the end of 24-week treatment was significantly higher in the group treated with a combination of miglitol plus sitagliptin than the two groups treated with miglitol alone and sitagliptin alone (Figure 2C). The majority of patients treated with sitagliptin alone and all patients treated with a combination of miglitol plus sitagliptin showed an increase in aGLP-1-iAUC (Figure 2C). The rate of aGIP-iAUC change from baseline to the end of 24-week treatment in the group treated with sitagliptin alone was significantly elevated compared with the two groups treated with miglitol alone and a combination with miglitol plus sitagliptin (Figure 2D). Furthermore, in all patients who received treatment with sitagliptin alone, except one patient, the rate of aGIP-iAUC change was increased (Figure 2D). The rate of glucose-iAUC change from baseline to the end of 24-week treatment in the group treated with a combination of miglitol plus sitagliptin was significantly lower than that in the group treated with sitagliptin (Figure 2E). The rate of IRI-iAUC change from baseline to the end of the 24-week treatment was significantly higher in the group treated with sitagliptin alone than the two groups treated with miglitol alone and a combination with miglitol plus sitagliptin (Figure 2F).

### 3.4. Correlation between Rates of aGLP-1-iAUC or aGIP-iAUC and SMI Changes

**Figure 3:**
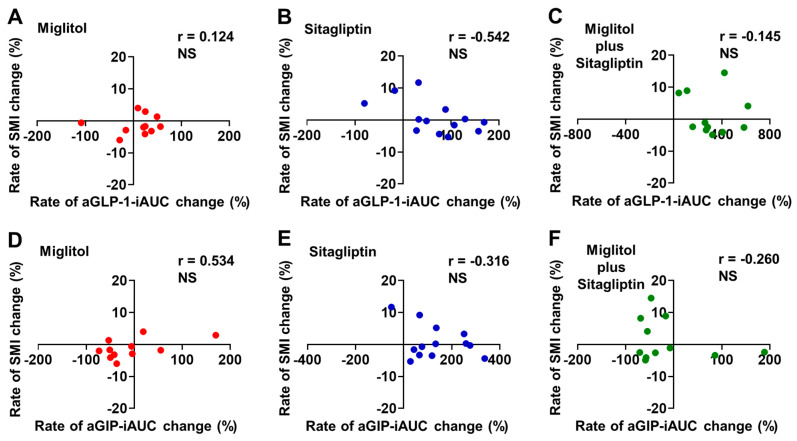
Correlation between rates of aGLP-1-iAUC or aGIP-iAUC and skeletal muscle mass index (SMI) changes from baseline to the end of 24-week treatment with miglitol, sitagliptin, and a combination with miglitol plus sitagliptin. (**A**,**D**) group treated with miglitol alone; (**B**,**E**) group treated with sitagliptin alone; (**C**,**F**) group treated with a combination of miglitol plus sitagliptin; NS, not significant.

The rate of aGLP-1-iAUC change from baseline to the end of the 24-week treatment was not significantly correlated with that of SMI change in all groups treated with miglitol, sitagliptin, and a combination with miglitol plus sitagliptin (Figure 3A–C). Similarly, a significant correlation between the rates of aGIP-iAUC and SMI changes from baseline to the end of 24-week treatment was not observed in all groups treated with miglitol, sitagliptin, and a combination with miglitol plus sitagliptin (Figure 3D–F).

### 3.5. Correlation between Rates of aGLP-1-iAUC or aGIP-iAUC and TBFMI Changes

**Figure 4:**
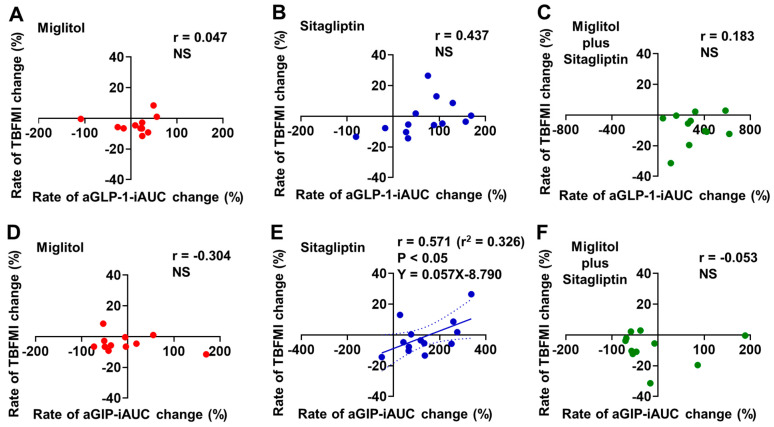
Correlation between rates of aGLP-1-iAUC or aGIP-iAUC and total body fat mass index (TBFMI) changes from baseline to the end of 24-week treatment with miglitol, sitagliptin, and a combination with miglitol plus sitagliptin. (**A**,**D**) group treated with miglitol alone; (**B**,**E**) group treated with sitagliptin alone; (**C**,**F**) group treated with a combination of miglitol plus sitagliptin; NS, not significant.

A significant correlation between the rates of aGLP-1-iAUC and TBFMI changes from baseline to the end of 24-week treatment was not found in the three groups treated with miglitol, sitagliptin, and a combination with miglitol plus sitagliptin (Figure 4A–C).The rate of aGIP-iAUC change from baseline to the end of 24-week treatment was positively correlated with that of TBFMI change in the sitagliptin treatment group (r = 0.571, *p* < 0.05, Figure 4E), whereas the two groups treated with miglitol alone and a combination with miglitol plus sitagliptin did not show a significant correlation between the rates of aGIP-iAUC and TBFMI changes from baseline to the end of 24-week treatment (Figure 4D,F).

### 3.6. Correlation between Rates of TBFMI and SMI Changes

**Figure 5:**
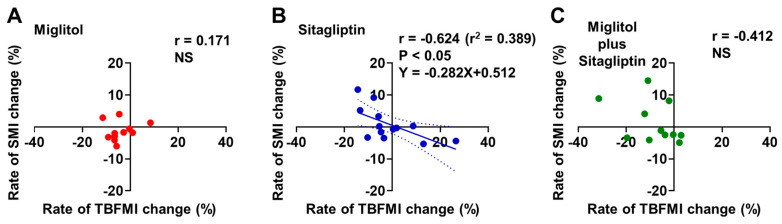
Correlation between rates of total body fat mass index (TBFMI) and skeletal muscle mass index (SMI) changes from baseline to the end of 24-week treatment with miglitol, sitagliptin, and a combination with miglitol plus sitagliptin. (**A**) group treated with miglitol alone; (**B**) group treated with sitagliptin alone; (**C**) group treated with a combination of miglitol plus sitagliptin; NS, not significant.

In the sitagliptin treatment group, the rate of TBFMI change from baseline to the end of the 24-week treatment was negatively correlated with that of SMI change (r = −0.624, *p* < 0.05, Figure 5B). No significant correlation was observed between the rates of TBFMI and SMI changes from baseline to the end of the 24-week treatment in the two groups treated with miglitol alone and a combination with miglitol plus sitagliptin (Figure 5A,C).

### 3.7. Correlation between Rates of IRI-iAUC and SMI or TBFMI Changes

**Figure 6:**
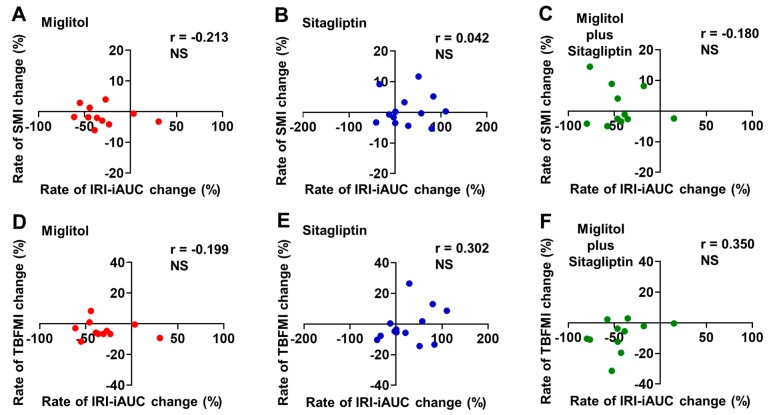
Correlation between rates of IRI-iAUC and skeletal muscle mass index (SMI) or total body fat mass index (TBFMI) changes from baseline to the end of 24-week treatment with miglitol, sitagliptin, and a combination with miglitol plus sitagliptin. (**A**,**D**) group treated with miglitol alone; (**B**,**E**) group treated with sitagliptin alone; (**C**,**F**) group treated with a combination of miglitol plus sitagliptin; NS, not significant.

Most of patients treated with miglitol alone and a combination with miglitol plus sitagliptin exhibited a reduction in the rate of IRI-iAUC change from baseline to the end of the 24-week treatment (Figure 6A,C,D,F), whereas more than half of patients treated with sitagliptin alone showed an increase in the rate of IRI-iAUC change (Figure 6B,E). The rate of IRI-iAUC change from baseline to the end of the 24-week treatment was not significantly correlated with that of SMI change and also that of TBFMI change in all groups treated with miglitol, sitagliptin, and a combination with miglitol plus sitagliptin (Figure 6A–F).

## 4. Discussion

In this secondary analysis of data in 35 T2D patients whose SMI values were available in the MASTER study, we first found that postprandial plasma aGLP-1 levels were highly elevated by the 24-week treatment with a combination of miglitol plus sitagliptin relative to their monotherapy when they were evaluated by using the rate of aGLP-1-iAUC change. A recent clinical study reported that GLP-1 infusion into the femoral artery enhanced postprandial muscle microvascular blood flow and augmented postprandial myofibrillar muscle protein synthesis in older people [21]. Furthermore, recent experimental studies have illustrated that a GLP-1 receptor agonist liraglutide administration increased citrate synthase activity and Cox5B expression, which represent mitochondrial content and are implicated in the regulation of muscle quality and volume in soleus muscle of spontaneously diabetic torii fatty rats [22], and that another GLP-1 receptor agonist exendin-4 ameliorated muscle atrophy by suppressing muscle atrophic factors and enhancing myogenic factors in a dexamethasone-induced mouse muscle atrophy model [23]. Although such possible crosstalk between GLP-1 infusion or GLP-1 receptor agonists and muscle regeneration has been reported, even plasma aGLP-1 elevation triggered by 24-week combination treatment with miglitol plus sitagliptin did not cause a significant increase in SMI in our study (Figure 1A–C). GLP-1 receptor agonists, so-called GLP-1 mimetics, are stable activators of the GLP-1 receptor, and they are also stabilized against DPP-4 [24]. Because of such pharmacokinetic properties, administration of GLP-1 receptor agonists could markedly elevate plasma concentrations of the GLP-1 mimetics, similar to plasma GLP-1 excursion during continuous GLP-1 infusion [25]. By contrast, DPP-4 inhibitors such as incretin or GLP-1 enhancers cause a modest elevation of postprandial plasma aGLP-1 [6]. As shown in the MASTER study, postprandial plasma aGLP-1 levels after 24-week combination treatment with miglitol plus sitagliptin were elevated to 11–14 pmol/L [19], whereas plasma concentrations of a GLP-1 receptor agonist (GLP-1 mimetic) liraglutide after its subcutaneous administration were demonstrated to be markedly increased to 4000–6000 pmol/L in T2D patients [25]. Given the evidence showing that GLP-1 receptors are expressed, to a lesser extent, in muscle tissues, further GLP-1 receptor signaling as provided by the GLP-1 receptor agonist administration may be needed to enhance muscle regeneration and increase skeletal muscle mass in T2D patients, and this idea may partly explain why the group treated with a combination of miglitol plus sitagliptin, which caused the highest plasma aGLP-1 excursion among the three treatment groups, failed to increase SMI. On the other hand, our study did not show the correlation between aGIP and SMI changes in three groups treated with miglitol, sitagliptin, and a combination with miglitol plus sitagliptin. To our knowledge, the relationship between GIP receptor signaling and skeletal muscle mass regulation has not been reported in both clinical and experimental studies, and therefore further studies would be needed to clarify the relationship.

Nevertheless, a recent retrospective observational study in a total of 105 T2D patients reported that an annual increase in SMI, albeit to a lesser extent, was observed in the patients treated with DPP-4 inhibitors compared with those without DPP-4 inhibitors [26]. On the other hand, our study did not show a 24-week increase in SMI in the two groups treated with DPP-4 inhibitor sitagliptin. In the above-mentioned retrospective observational study, 19% of the patients treated with DPP-4 inhibitors had sarcopenia [26], whereas there were no patients with sarcopenia in our study. Perhaps even a modest elevation of postprandial plasma aGLP-1 might be effective for enhancing muscle regeneration in case of patients with sarcopenia.

Similar to the findings regarding TBFM in the MASTER study [19], the current secondary analysis revealed that body fat accumulation was reduced by miglitol treatment when it was evaluated by using the values of TBFMI (Figure 1D–F). Possible relationships between αGIs such as miglitol and GIP secretion suppression [10,11,12,13] and also between the GIP receptor signaling enhancement and fat accumulation [27] have been well appreciated. Since the rates of aGIP-iAUC change were markedly lowered in the two groups treated with miglitol (Figure 2D), it is most likely that plasma aGIP reduction contributed to the suppression of body fat accumulation in these groups. Perhaps the long-term suppression of body fat accumulation would lead to a reduction in intramuscular fat accumulation, which is associated with loss of skeletal muscle mass and strength [28,29]. Indeed, the current data revealed a negative correlation between the rates of TBFMI and SMI changes in the sitagliptin group, which exhibited plasma aGIP elevation (Figure 5B). Hence, long-term miglitol treatment, which could continuously reduce plasma aGIP levels, might contribute to preventing the loss of skeletal muscle mass and strength via a reduction in intramuscular fat accumulation.

Another interesting finding in the current secondary analysis is that the rate of aGIP-iAUC change was positively correlated with that of TBFMI (Figure 4E) and the rate of TBFMI change was negatively correlated with that of SMI change (Figure 5B) in the sitagliptin group. As shown in the MASTER study, the sitagliptin treatment markedly increased serum insulin levels following meal ingestion, possibly due to elevated plasma levels of both aGLP-1 and aGIP [19]. It is well appreciated that excessive circulating insulin (i.e., hyperinsulinemia) is associated with body fat accumulation and obesity via inhibiting lipolysis as well as promoting lipogenesis in adipocytes [30,31]. Hence, more long-term postprandial plasma aGIP elevation in addition to plasma aGLP-1 augmentation by treatment with DPP-4 inhibitors might be involved in an increase in body fat mass through their additive insulinotropic effect, although the current secondary analysis did not show significant relationships between the rates of IRI-iAUC and SMI or TBFMI changes from baseline to the end of the 24-week treatment with sitagliptin alone (Figure 6A–F).

## 5. Study Limitations

There are several limitations in the current secondary analysis. First, there was no control group in the MASTER study. Second, the MASTER study was a pilot study performed with a small number of participants. As a result of the restricted sample size, the findings should be interpreted with caution. Third, food intake, which might affect the changes in body composition, was not examined in the MASTER study. Fourth, the patients taking metformin were included in the present study. Because a clinical study reported that metformin increased postprandial GLP-1 secretion in a small number of T2D patients [32], we cannot completely exclude the influence of metformin on postprandial plasma aGLP-1 excursion. Finally, the long-term effects of sitagliptin and/or miglitol treatment for more than one year on skeletal muscle mass regulation were not evaluated in the MASTER study.

## 6. Conclusions

The current secondary analysis revealed that postprandial plasma aGLP-1 and aGIP excursions modified by 24-week add-on treatment with an αGI miglitol and/or a DPP-4 inhibitor sitagliptin did not affect skeletal muscle mass regulation in T2D patients without sarcopenia. These findings indicate that αGIs and DPP-4 inhibitors could be used for gly-cemic management in T2D patients without adverse effects such as skeletal muscle mass reduction. Further GLP-1 receptor signaling as offered by treatment with GLP-1 receptor agonists may be needed to increase skeletal muscle mass in T2D patients without sarcopenia. Furthermore, the current study suggests that there are possible interrelationships among postprandial plasma aGIP excursion modified by sitagliptin, skeletal muscle mass, and body fat mass. Further long-term studies with a larger number of participants would be required to support the current findings.

## Figures and Tables

**Table 1:** Clinical characteristics and physiological and biochemical parameters at baseline and after 24-week treatment.

	Treatment Duration	Miglitol Group	Sitagliptin Group	Miglitol Plus Sitagliptin Group
*n* (male/female)		11 (9/2)	13 (10/3)	11 (6/5)
Age (years)		59.6 ± 7.5	60.5 ± 11.1	62.1 ± 11.4
Body weight (kg)	Baseline 24 weeks	80.7 ± 11.8 79.5 ± 12.4	75.7 ± 11.1 75.8 ± 11.8	72.0 ± 5.5 70.8 ± 6.4
Body mass index (kg/m^2^)	Baseline 24 weeks	28.9 ± 6.0 28.5 ± 6.2	28.7 ± 2.6 28.7 ± 2.6	28.2 ± 2.2 27.7 ± 2.6
Systolic blood pressure (mmHg)	Baseline 24 weeks	131 ± 18 124 ± 16	134 ± 19 127 ± 18	124 ± 7 122 ± 12
Diastolic blood pressure (mmHg)	Baseline 24 weeks	73 ± 11 72 ± 9	76 ± 12 76 ± 11	124 ± 7 122 ± 12
Fasting plasma glucose (mmol/L)	Baseline 24 weeks	7.4 ± 1.3 7.3 ± 1.4	7.6 ± 1.2 7.1 ± 1.1	7.9 ± 1.3 6.7 ± 1.1 *
Fasting serum IRI (pmol/L)	Baseline 24 weeks	34 ± 22 43 ± 25	58 ± 28 65 ± 34	60 ± 71 70 ± 105
HbA1c (%)	Baseline 24 weeks	6.9 ± 0.5 6.6 ± 0.6	7.4 ± 1.0 6.8 ± 0.6 **	7.1 ± 0.6 6.5 ± 0.4 *
LDL-cholesterol (mmol/L)	Baseline 24 weeks	3.22 ± 0.61 3.09 ± 0.62	3.26 ± 0.59 3.11 ± 0.62	3.46 ± 0.65 3.34 ± 0.81
HDL-cholesterol (mmol/L)	Baseline 24 weeks	1.47 ± 0.42 1.44 ± 0.33	1.31 ± 0.34 1.25 ± 0.28	1.38 ± 0.24 1.41 ± 0.29
Triglyceride (mmol/L)	Baseline 24 weeks	1.98 ± 1.23 1.70 ± 0.55	2.55 ± 1.58 2.37 ± 1.78	2.25 ± 2.09 1.61 ± 0.54
Fasting plasma active GLP-1 (pmol/L)	Baseline 24 weeks	1.56 ± 0.86 1.57 ± 0.72	1.78 ± 0.56 4.31 ± 2.95 *	1.42 ± 0.48 3.74 ± 2.86 **
Fasting plasma active GIP (pmol/L)	Baseline 24 weeks	7.6 ± 12.2 12.5 ± 8.0	12.2 ± 9.0 25.5 ± 18.7 **	6.3 ± 6.6 20.0 ± 14.9 *
Use of metformin (*n* [%])		6 (54.5)	4 (30.8)	4 (36.4)
Use of sulfonylurea (*n* [%])		2 (18.2)	1 (7.7)	3 (27.3)
Use of antihypertensive agents (*n* [%])		3 (27.3)	6 (46.2)	6 (54.5)
Use of lipid-lowering agents (*n* [%])		1 (9.1)	3 (23.1)	2 (18.2)

Values are means ± SD. * *p* < 0.01, ** *p* < 0.05 vs. Baseline.

## Data Availability

The datasets analyzed during the current study are not publicly available due to the lack of approval from our ethics committees.
